# Quantification of Naturally Occurring Prebiotics in Selected Foods

**DOI:** 10.3390/nu17040683

**Published:** 2025-02-14

**Authors:** Arianna Natale, Federica Fiori, Federica Turati, Carlo La Vecchia, Maria Parpinel, Marta Rossi

**Affiliations:** 1 Department of Clinical Sciences and Community Health, Dipartimento di Eccellenza 2023–2027, University of Milan, 20133 Milan, Italy; arianna.natale@unimi.it (A.N.); federica.turati@unimi.it (F.T.); carlo.lavecchia@unimi.it (C.L.V.); 2 Department of Medicine, University of Udine, 33100 Udine, Italy; federica.fiori@uniud.it

**Keywords:** prebiotics, galacto-oligosaccharides, fructo-oligosaccharides, food composition, database

## Abstract

*Background:* Prebiotics are non-digestible dietary compounds, defined as substrates that are utilised by host microorganisms conferring a health benefit. Although fructo-oligosaccharides (FOSs) and galacto-oligosaccharides (GOSs) are among the most studied prebiotics and support intestinal normobiosis, comprehensive data on their content in foods remain limited. *Objectives:* The objective was to quantify the content of FOSs (kestose, nystose, and 1 F-β-fructofuranosylnystose) and GOSs (raffinose and stachyose) in 35 foods, including fruit and nuts, legumes, and cereals. We also estimated the intakes of prebiotics in an Italian population. *Methods:* We analysed the prebiotic content in foods using high-performance anion-exchange chromatography with pulsed amperometric detection (HPAEC-PAD). We estimated the prebiotic intake of 100 healthy controls from a case-control study on colorectal cancer conducted in Italy between 2017 and 2019. We used dietary information collected through a food frequency questionnaire and the prebiotic data quantified in this and a previous study. *Results:* FOSs were mostly detected in cereal products, with wheat bran and whole-meal rye flour containing the highest amount (around 0.7 g/100 g each). GOSs were most abundant in legumes, especially in dried soy products (around 4.0 g/100 g each). Mean daily intake was 0.236 g for total FOSs and 0.371 g for total GOSs. Wheat bran, raspberries, chestnuts, walnuts, raisins, soy milk, and soy yoghurt overall accounted for 3.9% of kestose, 1.2% of nystose, 0% of 1F-β-fructofuranosylnystose, 15.5% of raffinose, and 8.3% of stachyose total intakes. *Conclusions:* The present study enables the development of a comprehensive database on prebiotic content in foods through a consistent analytical method. This makes prebiotic intake assessments more accurate than previously available data and facilitates future epidemiological studies investigating their potential effects on health.

## 1. Introduction

Prebiotics are non-digestible dietary compounds, defined as substrates that are selectively utilised by host microorganisms conferring health benefits [1]. Fructo-oligosaccharides (FOSs) and galacto-oligosaccharides (GOSs) are among the most studied prebiotics, known for their ability to promote the growth of intestinal bacteria (e.g., *Bifidobacteria* and *Lactobacilli*) that contribute to the maintenance of intestinal “normobiosis” [1,2,3]. As these oligosaccharides resist degradation by human digestive enzymes and gastric acidity, they are able to reach the colon intact, where they can exert their effects [1]. By enhancing intestinal barrier functions [4], GOSs and FOSs hold the potential for improving gut health [5], possibly playing a role in the management of several chronic diseases [6,7,8]. In clinical trials, prebiotic supplementations were linked to enhanced mineral absorption [9], immune system modulation [5], improved markers of metabolic syndrome [5], and reduced inflammatory markers such as IL-6 and IL-4 [10]. Prebiotics also have favourable effects on intestinal disorders [11,12] and were inversely associated with colorectal [13], laryngeal [14], and gastric [15] cancer risks. However, epidemiologic evidence remains scarce due to challenges to accurately measuring dietary prebiotic intake and the lack of a consistent and publicly accessible database on prebiotic content in foods. The availability of prebiotic contents in foods will allow for conducting new epidemiological studies that evaluate the influence of these compounds on different health outcomes.

Prebiotics occur in a variety of plant-based foods, such as fruits, vegetables, legumes, and grains, as reported by our previous study that quantified the prebiotic content of 78 foods [16]. The current study aims to expand those data by determining the content of FOSs (kestose, nystose, and 1 F-β-fructofuranosylnystose) and GOSs (raffinose and stachyose) in 35 additional foods, and to create a comprehensive and analytically consistent database. Hence, we estimated the prebiotic intake and the percentage of contribution of selected foods in a healthy population by applying the new updated prebiotic database to data from a food frequency questionnaire (FFQ) of an Italian case-control study.

## 2. Methods

We analysed the prebiotic content of 35 foods, grouped into three categories: 5 fruits (either fresh or dried) and nuts, 15 legumes and soy-based foods, and 15 cereal products. The foods were selected within food groups previously reported to contain prebiotics [13,16,17]. The selected foods were blueberries, raspberries, raisins, steamed chestnuts, and shelled dried walnuts for the fruit and nuts group; dried red adzuki beans, dried broad beans, shelled fresh broad beans, chickpea flour, fresh lupin beans, fresh soy sprouts, dried green mung beans, dried soybeans, toasted soybeans, soy flour, soy beverage (hereafter referred to as soy milk), dried soy textured vegetable protein (TVP)-based steak (from defatted soy flour), a soy-based alternative to yoghurt (hereafter referred to as soy yoghurt), tempeh, and tofu for the legume group; and amaranth (grains), buckwheat flour, bulgur, coarse-ground corn flour, shelled millet (grains), oat flour, quinoa (grains), basmati rice (grains), red rice (grains), “Venere” (black) rice (grains), whole-meal rye flour, semolina, spelt (grains), teff (grains), and wheat bran for the cereal group.

The analyses were carried out as in our previous research [16] by Neotron SpA, a food analysis company in Modena, Italy. Food products were purchased from Italian supermarkets and mass retailers between March and April 2024. They were homogenised and stored at −20 °C until the analyses took place. The contents of kestose, nystose, 1 F-β-fructofuranosylnystose, raffinose, and stachyose were determined using alkaline hydrolysis coupled with high-performance anion-exchange chromatography with pulsed amperometric detection (HPAEC-PAD). Quantification limits for prebiotics ranged from 0.002 g to 0.05 g per 100 g, varying by food type. The methodology is linear (linearity, R2 *>* 0.99 for all compounds), precise (precision, RSD% *<* 10% for all compounds), and accurate (recovery, 87–101%).

We estimated the prebiotic intake in the control population from a case-control study on colorectal cancer (CRC) conducted in Milan, Italy, between 2017 and 2019 [18]. All participants underwent a colonoscopy that allowed the identification of 100 healthy controls, i.e., participants with a confirmed absence of both intestinal adenomas and CRC. The habitual diet was evaluated using a reproducible [19] and valid [17] FFQ, which included questions on the weekly consumption frequencies of 81 FFQ items (75 food and 6 alcoholic drink items). From this information, we calculated the individual daily prebiotic intake using data from previous [16] and new estimates on the prebiotic content in foods. Foods with prebiotic content below the detection limit (trace amounts) were set to zero. Out of the 81 FFQ items, the prebiotic content was available for 42 FFQ items. Of these, data on 37 FFQ items were derived from the previous estimates and on 5 FFQ items from the new estimates, including 9 foods: raspberries; steamed chestnuts, dried and shelled walnuts and raisins; soy milk and soy yoghurt; tofu and tempeh; and wheat bran. We then analysed the distribution of prebiotic intakes, and we determined the mean percentage contributions of these 5 newly analysed FFQ items to the total prebiotic intake.

## 3. Results

Table 1 gives the content of FOSs and GOSs in the 35 analysed foods. In the fruits and nuts group, kestose was detected in steamed chestnuts and raspberries (0.0152 g/100 g and 0.0159 g/100 g, respectively); nystose was present in blueberries (0.0106 g/100 g). Meanwhile, 1 F-β-fructofuranosylnystose was undetectable in all fruits and nuts. FOSs were undetectable in all legume products, except for kestose in toasted soybeans (0.0031 g/100 g). FOSs were mostly represented in cereal products. Kestose was detected mostly in wheat bran (0.7300 g/100 g), while nystose and 1 F-β-fructofuranosylnystose were in whole-meal rye flour (0.5010 g/100 g and 0.1080 g/100 g, respectively).

GOSs from fruits and nuts were detected in steamed chestnuts (0.1190 g/100 g for raffinose, 0.1100 g/100 g for stachyose), in raisins (0.0545 g/100 g for raffinose, stachyose <0.01 g/100 g) and in dried shelled walnuts (0.0870 g/100 g for raffinose and 0.0470 g/100 g for stachyose). GOSs were most abundant in legume products, with non-detectable amounts only in fresh products, such as broad beans, lupin beans, and soy sprouts. The highest amount of raffinose was contained in toasted soybeans (0.7010 g/100 g) and that of stachyose in TVP-based steak (3.6400 g/100 g). Raffinose was detectable in all cereal products, with wheat bran containing the highest (1.2100 g/100 g) and amaranth grains containing the lowest amounts (0.0023 g/100 g). Stachyose was detected in amaranth grains (0.2120 g/100 g), shelled millet grains (0.0107 g/100 g), oat flour (0.1640 g/100 g), quinoa grains (0.0464 g/100 g), and wheat bran (0.0213 g/100 g).

Table 2 gives the distribution of dietary prebiotic intake. The mean intake of total FOSs was 0.236 g/die, with a standard deviation (SD) of 0.100 g/die. Kestose was the most abundant FOS, with a mean of 0.209 g/die (SD: 0.095 g/die), followed by nystose, with a mean of 0.019 g/die (SD: 0.010 g/die), and 1 F-β-fructofuranosylnystose, with a mean of 0.007 g/die (SD: 0.006 g/die). For the total GOSs, the mean intake was 0.371 g/die (SD: 0.218 g/die). Stachyose was the most abundant GOS, with a mean of 0.242 g/die (SD: 0.186 g/die). The mean intake of raffinose was 0.128 g/die (SD: 0.053 g/die).

Table 3 reports the mean percentage of contribution of the five food items included in our FFQ. The consumption of these five items contributed 3.9% (mostly wheat bran: 3.5%) to kestose intake, 1.2% (completely wheat bran: 1.2%) to nystose intake, 15.5% (mostly wheat bran: 8.8%) to raffinose intake, and 8.3% (mostly soy milk and soy yoghurt: 6.6%) to stachyose intake. Concerning 1 F-β-fructofuranosylnystose, a null contribution was found from these foods.

## 4. Discussion

Our novel results on the prebiotic content of 35 foods, including fruits, nuts, legumes, and cereals, integrate data from previously analysed foods [16] and lead to the creation of an extensive database of the prebiotic content of 113 foods. Of all the foods analysed, cereals, such as wheat bran and whole-meal rye flour, and root vegetables were the richest in FOSs. Legumes, especially dried soy products, were the richest in GOSs.

These data will serve as a resource for future studies investigating the relationship between prebiotic intake and health outcomes. To this aim, these data will be added to the Food Composition Database for Epidemiological Studies in Italy, which is a compiled, publicly available database (at https://bda.ieo.it/, accessed on 20 December 2024) [20].

To quantify the prebiotic content in foods, we used the HPAEC-PAD method, which overcomes the limitations of conventional high-performance liquid chromatography (HPLC) in oligosaccharide analysis [21,22,23]. For both FOSs [22] and GOSs [24], HPAEC-PAD has been recognised among the highest sensitive detection methods, allowing for reaching low quantification limits and detecting prebiotics over a broader polymerisation range.

Until now, methods for quantifying prebiotic content in foods have been inconsistent and data have been scarce [16,25,26,27,28,29,30]. This makes it difficult to compare the available data with our data. To the best of our knowledge, 20 of the 35 foods we looked at had no data on the prebiotic content we analysed.

Sugar content tables from the United States Department of Agriculture [31] provide information on the raffinose and stachyose content of raw adzuki beans, raw broad beans, raw mung beans, raw soybeans, soy flour, amaranth grains, millet grains, and wheat bran. However, these tables are no longer in active use and could not be suitable to be used as a benchmark for later results [31].

Among the fruits studied, blueberries and raspberries were analysed for FOS content [25,26], with different results. Muir et al. used HPLC with evaporative light-scattering detectors (ELSD) and reported 0.14 g/100 g of kestose and 0.30 g/100 g of nystose in blueberries and 0.08 g/100 g of kestose and 0.22 g/100 g of nystose in raspberries [25]. Campbell et al. used HPLC with a pulsed electrochemical detector (PED) and found 0.02 g/100 g of kestose and 0.01 g/100 g of nystose in blueberries and 0.14 g/100 g of kestose and 0.01 g/100 g of nystose in raspberries, as well as no 1 F-β-fructofuranosylnystose in either berry [26]. In our analysis, only nystose for blueberries and kestose for raspberries were detected. Our results for nystose in blueberries (0.0106 g/100 g) corresponded to the 3.5% found by Muir et al. [25] and were almost identical to Campbell et al. [26], while in raspberries (0.0159 g/100 g), we found approximately 20% of kestose content reported by Muir et al. [25] and 11% of that reported by Campbell et al. [26].

Some studies have quantified the prebiotic content of legumes using dry-matter analysis with HPLC with refractive index (RI) [27,28,29]. A comparison of those with our results, which are based on whole food matrices may introduce bias. Martinez-Villanuenga et al. [27] reported the GOS content of 13 varieties of lupins (on dry matter), including several cultivars of the Italian lupin (*Lupinus albus* L.): the raffinose content ranged from 0.33% to 0.62% and stachyose from 4.98% to 7.26%. Wang et al. [28] and Kaczmarska et al. [29] quantified GOSs in soy products (dry-weight bases). Wang et al. [28] reported that raw soybeans contained 0.752 g/100 g of raffinose and 4.13 g/100 g of stachyose, while soy milk had 0.687 g/100 g of raffinose and 3.79 g/100 g of stachyose. Kaczmarska et al. [29] found higher concentrations of GOSs compared to Wang et al. [28], with raw soybeans (referred to as raw soy) containing 1.89 g/100 g of raffinose and 13.27 g/100 g of stachyose. In soy flour, Kaczmarska et al. reported 3.19 g/100 g of raffinose and 14.10 g/100 g of stachyose [29].

Regarding cereal products, data on the FOS content in wheat bran and rye dark flour were determined using HPAEC-PAD, allowing for a direct comparison with our findings [30]. Their results showed that wheat bran contained 0.50 g/100 g of kestose, 0.03 g/100 g of nystose, and <0.02 g/100 g of 1 F-β-fructofuranosylnystose. For whole-meal rye flour, the levels were 0.58 g/100 g of kestose, 0.33 g/100 g of nystose, and 0.26 g/100 g of 1 F-β-fructofuranosylnystose. In wheat bran, we found around 1.5 times the kestose and the same amount of nystose as Hogarth et al. [30]. Neither analysis detected 1 F-β-fructofuranosylnystose in wheat bran. For whole-meal rye flour, we found the 86% of kestose (0.5010 g/100 g), 50% of nystose (0.1650 g/100 g), and 41% of 1 F-β-fructofuranosylnystose (0.1080 g/100 g) reported by Hogarth et al. [30]. Given the comparable analytical method, the variations are likely due to differences in the cereal products analysed, since they may have had different bran-to-endosperm ratios or may have been processed differently. 

The foods analysed in the present study are relevant components of modern diets [32]. The consumption of plant-based dairy substitutes, of which soy-based products represent a great part [33,34], has risen in Western countries over the 2000s [35,36]. In Italian adults, the percentage of dairy-alternative consumers rose from 0.4% in 2005 to 9.2% in 2018 [37]. Comparable trends were found across Europe (e.g., in Dutch adults: 2.5% in 2007 vs. 11.1% in 2012; in UK adults: 3.9% in 2000 vs. 6.0% in 2008) [37]. Soy-based foods positively influence several health outcomes, including a decrease in obesity/overweight [38], reduction in cancer risk [39], and cardiovascular risk [40], in addition to total mortality [41]. A protective effect of legume consumption on overall mortality [42], coronary heart disease [42], selected cancers [43], and type-2 diabetes [44] has also been reported. 

We included in our databases a wide range of legumes and cereals to take into account the growing interest in both traditional and non-traditional varieties in Italy. The inclusion of foods such as lupins, fava beans, mung beans, adzuki beans, different types of rice, millet, quinoa, and nuts and dried fruits, which are traditionally found in the diets of different populations, broadens the applicability of our findings [45,46]. Although the prebiotic content of these foods may vary between geographical regions due to differences in cultivars, climate, and cultivation techniques [47], our data provide valuable information, considering the absence of region-specific databases on the prebiotic content of foods.

Our estimates among the control group of a case-control study on CRC align with a previous assessment of prebiotic consumption in controls from another Italian CRC case-control study [13], although our data indicate somewhat higher intakes. We observed an increase in the mean intake of both FOSs (+20%) and GOSs (+12%) compared to the earlier study. This is largely due to the inclusion of prebiotic-rich foods, such as wheat bran and soy products, which contributed up to 15% of the prebiotic intake in our population. The identification of contributors to dietary prebiotic intake highlights the importance of considering these foods in prebiotic intake assessments.

Prebiotics play a fundamental role in shaping gut microbiota against dysbiosis [48], which is involved in the regulation of inflammatory and metabolic pathways [2,49]. Dysbiosis has been associated with cardiovascular and gut inflammatory diseases, as well as digestive tract cancers [18,49,50,51]. Many foods rich in prebiotics have also been associated with a reduced risk of these diseases [52,53,54,55]. Nuts, fruit, and cereals (mainly unrefined) are an essential part of the Mediterranean diet and contribute to its beneficial health effects, including the risk reduction of cancer [42], and metabolic and cardiovascular diseases [56]. A deeper understanding of the association between prebiotics and health outcomes might influence dietary guidelines or public health interventions.

## 5. Conclusions

To date, the literature on the prebiotic content in foods reports partial results on specific food types, obtained with heterogeneous analytical methods. The consistent application of a highly sensitive detection technique ensures the reliability of our results. Our data will facilitate more accurate assessments of prebiotic intake, allowing future epidemiological studies to evaluate the role of prebiotics on health.

## Figures and Tables

**Table 1:** Naturally occurring fructo-oligosaccharides and galacto-oligosaccharides content (g/100 g) in a selection of foods. Italy, 2024.

	FOSs			GOSs	
Common Name	Kestose	Nystose	1 F-β-FFnystose	Raffinose	Stachyose
Fruits and nuts					
Blueberries	<LOQ ^a^	0.0106	<LOQ ^a^	<LOQ ^b^	<LOQ ^b^
Chestnut, steamed	0.0152	<LOQ ^a^	<LOQ ^c^	0.1190	0.1100
Raisins	<LOQ ^b^	<LOQ ^d^	<LOQ ^d^	0.0545	<LOQ ^b^
Raspberries	0.0159	<LOQ ^a^	<LOQ ^c^	<LOQ ^a^	<LOQ ^c^
Walnuts, shelled, dried	<LOQ ^a^	<LOQ ^a^	<LOQ ^a^	0.0870	0.0470
Legume products					
Adzuki beans, red, dried	<LOQ ^e^	<LOQ ^e^	<LOQ ^e^	0.1240	2.7000
Broad beans, dried, shelled	<LOQ ^a^	<LOQ ^c^	<LOQ ^a^	0.1710	0.5880
Broad beans, fresh	<LOQ ^b^	<LOQ ^b^	<LOQ ^b^	<LOQ ^b^	<LOQ ^b^
Chickpeas flour	<LOQ ^b^	<LOQ ^b^	<LOQ ^a^	0.4730	1.6400
Lupin beans, fresh	<LOQ ^a^	<LOQ ^a^	<LOQ ^a^	<LOQ ^a^	<LOQ ^a^
Mung beans, green, dried	<LOQ ^a^	<LOQ ^a^	<LOQ ^a^	0.3280	1.5500
Soybeans, toasted	0.0031	<LOQ ^b^	<LOQ ^b^	0.7010	3.0700
Soybeans, dried	<LOQ ^d^	<LOQ ^a^	<LOQ ^a^	0.5130	2.8100
Soy flour	<LOQ ^e^	<LOQ ^e^	<LOQ ^a^	0.6770	3.2200
Soy milk	<LOQ ^a^	<LOQ ^a^	<LOQ ^a^	0.0389	0.2300
Soy sprouts, fresh	<LOQ ^a^	<LOQ ^a^	<LOQ ^a^	<LOQ ^c^	<LOQ ^a^
Soy TVP-based steak, dried	<LOQ ^b^	<LOQ ^b^	<LOQ ^b^	1.1700	3.6400
Soy yogurt	<LOQ ^a^	<LOQ ^b^	<LOQ ^a^	0.0263	0.1120
Tempeh	<LOQ ^b^	<LOQ ^b^	<LOQ ^b^	<LOQ ^b^	0.0349
Tofu	<LOQ ^b^	<LOQ ^b^	<LOQ ^a^	0.0263	0.1140
Cereal products					
Amaranth (grains)	0.0279	<LOQ ^a^	<LOQ ^a^	0.6880	0.2120
Buckwheat flour	0.0032	<LOQ ^a^	<LOQ ^a^	0.0023	<LOQ ^a^
Bulgur	0.2740	0.0197	<LOQ ^e^	0.3190	<LOQ ^e^
Coarse-ground corn flour	<LOQ ^a^	<LOQ ^a^	<LOQ ^a^	0.0060	<LOQ ^a^
Millet, shelled (grains)	0.0040	<LOQ ^a^	<LOQ ^a^	0.0535	0.0107
Oat flour	0.0038	<LOQ ^c^	<LOQ ^a^	0.1010	0.1640
Quinoa (grains)	<LOQ ^c^	<LOQ ^b^	<LOQ ^b^	0.0569	0.0464
Rice, Basmati (grains)	<LOQ ^a^	<LOQ ^a^	<LOQ ^a^	0.0090	<LOQ ^a^
Rice, red (grains)	0.0023	<LOQ ^a^	<LOQ ^a^	0.0486	<LOQ ^a^
Rice, Venere (grains)	<LOQ ^a^	<LOQ ^a^	<LOQ ^a^	0.0862	<LOQ ^a^
Rye flour, whole-meal	0.5010	0.1650	0.1080	0.3070	<LOQ ^b^
Semolina	0.1950	<LOQ ^d^	<LOQ ^d^	0.2370	<LOQ ^d^
Spelt (grains)	0.3710	0.0194	<LOQ ^e^	0.4090	<LOQ ^f^
Teff (grains)	<LOQ ^a^	<LOQ ^a^	<LOQ ^a^	0.1410	<LOQ ^a^
Wheat bran	0.7300	0.0234	<LOQ ^e^	1.2100	0.0213

FOSs: fructo-oligosaccharides; GOSs: galacto-oligosaccharides, 1 F-β-FFnystose: 1 F-β-fructofuranosylnystose; LOQ: limit of quantification; TVP: textured vegetable protein. ^a^ LOQ = 0.0020; ^b^ LOQ = 0.0100; ^c^ LOQ = 0.0050; ^d^ LOQ = 0.0500; ^e^ LOQ = 0.0200; ^f^ LOQ = 0.1000.

**Table 2:** Dietary prebiotic intake (g/day) in a population of 100 healthy controls from a case-control study. Italy, 2017–2019.

	Mean	SD	Median (25th–75th)
Total FOSs	0.236	0.100	0.217 (0.168–0.278)
Kestose	0.209	0.095	0.190 (0.142–0.247)
Nystose	0.019	0.010	0.017 (0.013–0.023)
1 F-β-FFnystose	0.007	0.006	0.009 (0.001–0.011)
Total GOSs	0.371	0.218	0.339 (0.227–0.481)
Raffinose	0.128	0.053	0.119 (0.091–0.161)
Stachyose	0.242	0.186	0.202 (0.099–0.340)

SD: standard deviation; FOSs: fructo-oligosaccharides; 1 F-β-FFnystose: 1 F-β-fructofuranosylnystose; GOSs: galacto-oligosaccharides.

**Table 3:** Mean percentage of contribution of selected foods to prebiotic intake in a population of 100 healthy controls from a case-control study. Italy, 2017–2019.

	FOSs		GOSs	
	Kestose	Nystose	Raffinose	Stachyose
Raspberries (%)	0.28	0.00	0.00	0.00
Chestnuts, walnuts and raisins (%)	0.17	0.00	4.34	1.37
Soy milk and soy yoghurt (%)	0.00	0.00	2.26	6.59
Tofu and tempeh (%)	0.00	0.00	0.09	0.26
Wheat bran (%)	3.45	1.21	8.77	0.08
Total (%)	3.90	1.21	15.46	8.30

FOSs: fructo-oligosaccharides; GOSs: galacto-oligosaccharides.

## Data Availability

The data presented in this study are available upon request from the corresponding author.
